# Nucleus accumbens shell modulates seizure propagation in a mouse temporal lobe epilepsy model

**DOI:** 10.3389/fcell.2022.1031872

**Published:** 2022-12-15

**Authors:** Wenjie Zou, Zhipeng Guo, Longge Suo, Jianping Zhu, Haiyang He, Xiufeng Li, Kewan Wang, Rongqing Chen

**Affiliations:** ^1^ Guangdong Province Key Laboratory of Psychiatric Disorders, Department of Neurobiology, School of Basic Medical Sciences, Southern Medical University, Guangzhou, China; ^2^ Department of Neurosurgery, Nanfang Hospital, Southern Medical University, Guangzhou, China; ^3^ Key Laboratory of Mental Health of the Ministry of Education, Guangdong-Hong Kong-Macao Greater Bay Area Center for Brain Science and Brain-Inspired Intelligence, Southern Medical University, Guangzhou, China

**Keywords:** epilepsy, temporal lobe epilepsy, nucleus accumbens, medium spiny neurons, dopamine receptor

## Abstract

Temporal lobe epilepsy (TLE) is the most common form of epilepsy with focal seizures which in some conditions can develop into secondarily generalized tonic–clonic seizures by the propagation of epileptic activities in the temporal lobe to other brain areas. The nucleus accumbens (NAc) has been suggested as a treatment target for TLE as accumulating evidence indicates that the NAc, especially its shell, participates in the process of epileptic seizures of patients and animal models with TLE. The majority of neurons in the NAc are GABAergic medium spiny neurons (MSNs) expressing dopamine receptor D1 (D1R) or dopamine receptor D2 (D2R). However, the direct evidence of the NAc shell participating in the propagation of TLE seizures is missing, and its cell type-specific modulatory roles in TLE seizures are unknown. In this study, we microinjected kainic acid into basolateral amygdala (BLA) to make a mouse model of TLE with initial focal seizures and secondarily generalized seizures (SGSs). We found that TLE seizures caused robust c-fos expression in the NAc shell and increased neuronal excitability of D1R-expressing MSN (D1R-MSN) and D2R-expressing MSN (D2R-MSN). Pharmacological inhibition of the NAc shell alleviated TLE seizures by reducing the number of SGSs and seizure stages. Cell-type-specific chemogenetic inhibition of either D1R-MSN or D2R-MSN showed similar effects with pharmacological inhibition of the NAc shell. Both pharmacological and cell-type-specific chemogenetic inhibition of the NAc shell did not alter the onset time of focal seizures. Collectively, these findings indicate that the NAc shell and its D1R-MSN or D2R-MSN mainly participate in the propagation and generalization of the TLE seizures.

## Introduction

Epilepsy is a major neurological disorder in which excessive electrical excitability originates from some clusters of brain neurons in the epileptic focus and triggers epileptic seizures. Initial abnormal excitability may be quenched by increased local inhibition and thereby causes only focal seizures, while uncontrolled focal excitability or generalized onset of over excitability propagates hypersynchronously to many brain structures and thereby causes generalized seizures. Temporal lobe epilepsy (TLE) is the most common type of epilepsy as it affects more than 60% of patients with epilepsy. While TLEs are focal epilepsy, the TLEs with secondarily generalized seizures (SGSs) contribute to a large number of severe seizure-associated injury, neurological degradation, and sudden death (Kodankandath et al., 2022). Preventing seizure propagation and generalization are important to limit severity of seizures, especially for drug-resistant seizures.

The nucleus accumbens (NAc) is a structure within ventral stratum, composed of core and shell substructures. Nearly 95% of NAc neurons are GABAergic medium spiny neurons (MSNs) receiving not only dopaminergic inputs from the ventral tegmental area (VTA), but also excitatory inputs from the hippocampus, amygdala, prefrontal cortex, and thalamus (Sundar et al., 2021). The MSNs express either dopamine D1 receptor (D1R) or D2 receptor (D2R). Both D1R-expressing MSN (D1R-MSN) and D2R-expressing MSN (D2R-MSN) are GABAergic projection cells biasedly innervating the VTA, basal ganglia, thalamus, etc. (Xu et al., 2020). On the basis of these anatomic and circuit connections, D1R-MSN and D2R-MSN in the NAc participate in motivational, rewarding, and emotional processing, and limbic-motor integration. Consequently, structural and functional abnormalities of the NAc lead to not only neuropsychiatric diseases such as addiction, depression, autism, schizophrenia, and obsessive-compulsive disorder, but also neurological diseases such as motor dysfunction (Salgado and Kaplitt, 2015).

Accumulating literatures show that NAc, especially the shell, participates in epileptic seizures of patients with TLE or animal models of TLE by observing its seizure-related pathological changes in neuronal activity (Buckby and Lacey, 2001; Long et al., 2009), metabolism (Loscher et al., 1996), structural morphology, and functional connectivity (Peng et al., 2014; Zhao et al., 2018; Yang et al., 2020). By using functional mapping and detecting electrophysiological activity and product of metabolism in the NAc during and after TLE seizures, some studies indicate that the NAc involves in the propagation of seizure activity of rat models of TLE (Lothman et al., 1985; Loscher et al., 1996; Pereira de Vasconcelos et al., 1999). Moreover, it has been reported that seizures are modulated by the activations of dopaminergic receptors. Systematic pharmacological activation of D1R is proconvulsant (O’Sullivan et al., 2008) and that of D2R displays antiepileptic effects in adult animal models (Brodovskaya and Kapur, 2021). Interestingly, Wahnschaffe and Loscher reported that administration of D2R agonist LY 171555 into the NAc was enough to provide antiepileptic effects in the amygdala-kindled epilepsy model (Wahnschaffe and Loscher, 1991). Therefore, NAc, especially the shell, and its dopaminergic systems have been considered as therapeutic targets of anti-epileptic treatments with drugs or deep brain stimulation (DBS) (Kowski et al., 2015). However, the direct evidence of the NAc shell and its cell type-specific modulatory roles in TLE seizures are missing. In this research, we used a mouse model of TLE of which the seizure onset zone is within the basolateral amygdala (BLA) and investigated whether the NAc shell and its D1R-MSN and D2R-MSN modulate TLE seizures.

## Results

### NAc shell is activated in the process of TLE

**FIGURE 1:**
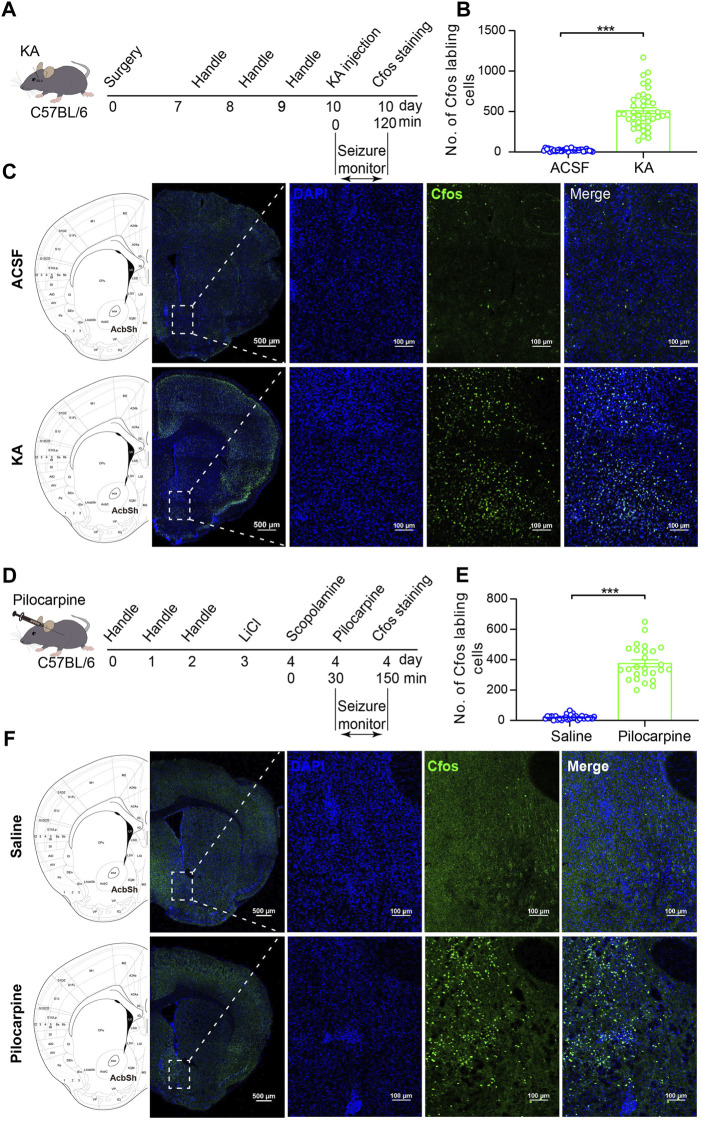
C-fos expression in the NAc shell cells after TLE seizures. **(A)** Schematic diagram of TLE seizure induction on the C57BL/6 mouse by intra-basolateral amygdala (intra-amygdala) microinjection of KA. **(B)** Quantification of the c-fos expression in the NAc shell after seizures of the intra-amygdala KA model of TLE. ACSF group: *n* = 33 slices from four mice; KA group: *n* = 40 slices from four mice; ***p < 0.001, Student’s unpaired t test. **(C)** Representative confocal images showing the c-fos expression in the NAc shell after seizures of the intra-amygdala KA model of TLE. **(D)** Schematic diagram of mouse TLE seizure induction by i.p. Injection of LiCl–pilocarpine. **(E)** Quantification of the c-fos expression in the NAc shell after seizures of the systematic pilocarpine model of TLE. Saline group: *n* = 30 slices from four mice; pilocarpine group: n = 26 slices from three mice; ***p < 0.001, Student’s unpaired t test. **(F)** Representative confocal images showing the c-fos expression in the NAc shell after seizures of the systematic pilocarpine model of TLE.

Intra-amygdala stereotaxic microinjection of kainic acid (KA) is a well-established model of TLE with SGSs (Ben-Ari et al., 1980). We microinjected KA into mouse BLA to induce TLE, a model of epilepsy consisting of focal seizures (stage 1–3 of Racine’s scale) and SGSs (stage 4–6 of Racine’s scale) (Racine, 1972a; b). To see whether the NAc is involved in intra-amygdala KA-induced TLE seizures, we used immunofluorescence staining to detect the expression of c-fos which is an anatomic marker of acute neuronal activity 2 h after seizure induction (Figure 1A). We found robust c-fos expression in the widespread cortex and some subcortical structures including the NAc shell and core of the epileptic mice as is shown in the sampling images (Figure 1C). For the NAc, recent clinical and animal data indicate that the shell substructure might mainly participate in epilepsy (de Oliveira et al., 2019; Yang et al., 2020). We thereafter focused on the roles of NAc shell in the involvement in TLE seizures.

Our immunostaining data showed that the c-fos expression was significantly higher in the NAc shell of the epileptic animals, compared with vehicle control animals (Figures 1B,C). To see whether this is a common phenomenon in the TLE models, we detected the c-fos expression in the NAc shell in another model of TLE which was induced by intraperitoneal (i.p.) injection of LiCl–pilocarpine (Figure 1D). In this model, the c-fos expression was also significantly higher in the NAc shell of the epileptic animals comparing with the vehicle control animals (Figures 1E,F). These data show that the NAc shell is activated in the process of TLE, indicating the involvement of the NAc shell in the TLE.

Although both models are well-established TLE models with convulsive status epilepticus (SE) manifested as generalized tonic–clonic seizures, the intra-amygdala KA model differs from i.p. injection of the LiCl–pilocarpine model in that its epileptic initiation site is appointed at the BLA and the abnormal activity of other brain region such as the NAc shell is due to seizure propagation. Thus, in the following experiments, we continued using the intra-amygdala induction of TLE seizures to investigate the roles of NAc shell and its MSNs in the modulation of propagation of TLE seizures.

### Both D1R-MSN and D2R-MSN in the NAc shell are activated in the process of TLE

**FIGURE 2:**
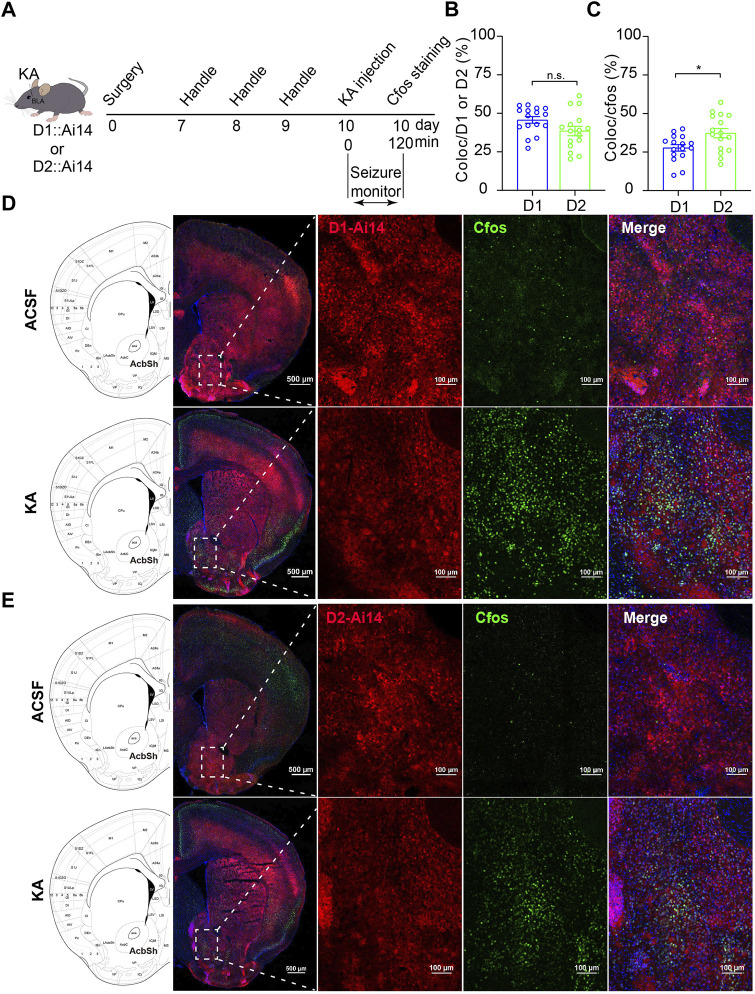
C-fos expression of the MSNs in the NAc shell after TLE seizures. **(A)** Schematic diagram of TLE seizure induction on D1R-cre::Ai14 or D2R-cre::Ai mouse by intra-basolateral amygdala (intra-amygdala) microinjection of KA. **(B)** Quantification of the percentage of c-fos^+^ cells in D1R-MSNs or D2R-MSNs (tdTomato^+^ from Ai14) in the NAc shell. Coloc indicates c-fos^+^ and tdTomato^+^ cells; D1/2 indicates tdTomato^+^ D1R-MSNs or D2R-MSNs. D1 group: *n* = 16 slices from two mice; D2 group: *n* = 16 slices from two mice; *p* > 0.05, Student’s unpaired t test. **(C)** Quantification of the percentage of tdTomato^+^ D1R-MSNs or D2R-MSNs in c-fos^+^ cells in the NAc shell. D1 group: *n* = 16 slices from two mice; D2 group: *n* = 16 slices from two mice; *p < 0.05, Student’s unpaired t test. **(D)** Representative confocal images showing the c-fos expression in the NAc shell of D1R-cre::Ai14 mouse after seizures of the intra-amygdala KA model of TLE. **(E)** Representative confocal images showing the c-fos expression in the NAc shell of D2R-cre::Ai14 mouse after seizures of the intra-amygdala KA model of TLE.

Next, to know which type of MSNs in the NAc shell is involved in the process of TLE, we performed TLE induction by intra-amygdala KA microinjection to D1::Ai14 (D1R-cre::Ai14) and D2::Ai14 (D2R-cre::Ai14) mice whose D1R-MSN and D2R-MSN express robust tdTomato fluorescence due to Cre-mediated recombination (Figure 2A). To detect seizure-associated cell-type-specific activation of MSNs, c-fos staining was performed 2 h after seizure induction and the colocalization of c-fos with the reporter fluorescence of D1R-MSN or D2R-MSN was analyzed. This analysis showed that the percentage of D1R-MSN-expressed c-fos was similar to that of D2R-MSN-expressed c-fos (D1R-MSN: 45.73 ± 2.16%, *n* = 16; D2R-MSN: 38.54 ± 3.49%, *n* = 14; *p* > 0.05. Figures 2B,D,E), while the proportion of D1R-MSNs was relatively less than that of D2R-MSNs in the total cells expressing c-fos (D1R-MSN: 27.69 ± 2.13%, *n* = 16; D2R-MSN: 37.30 ± 3.43%, *n* = 14. *p* < 0.05. Figures 2C–E).

**FIGURE 3:**
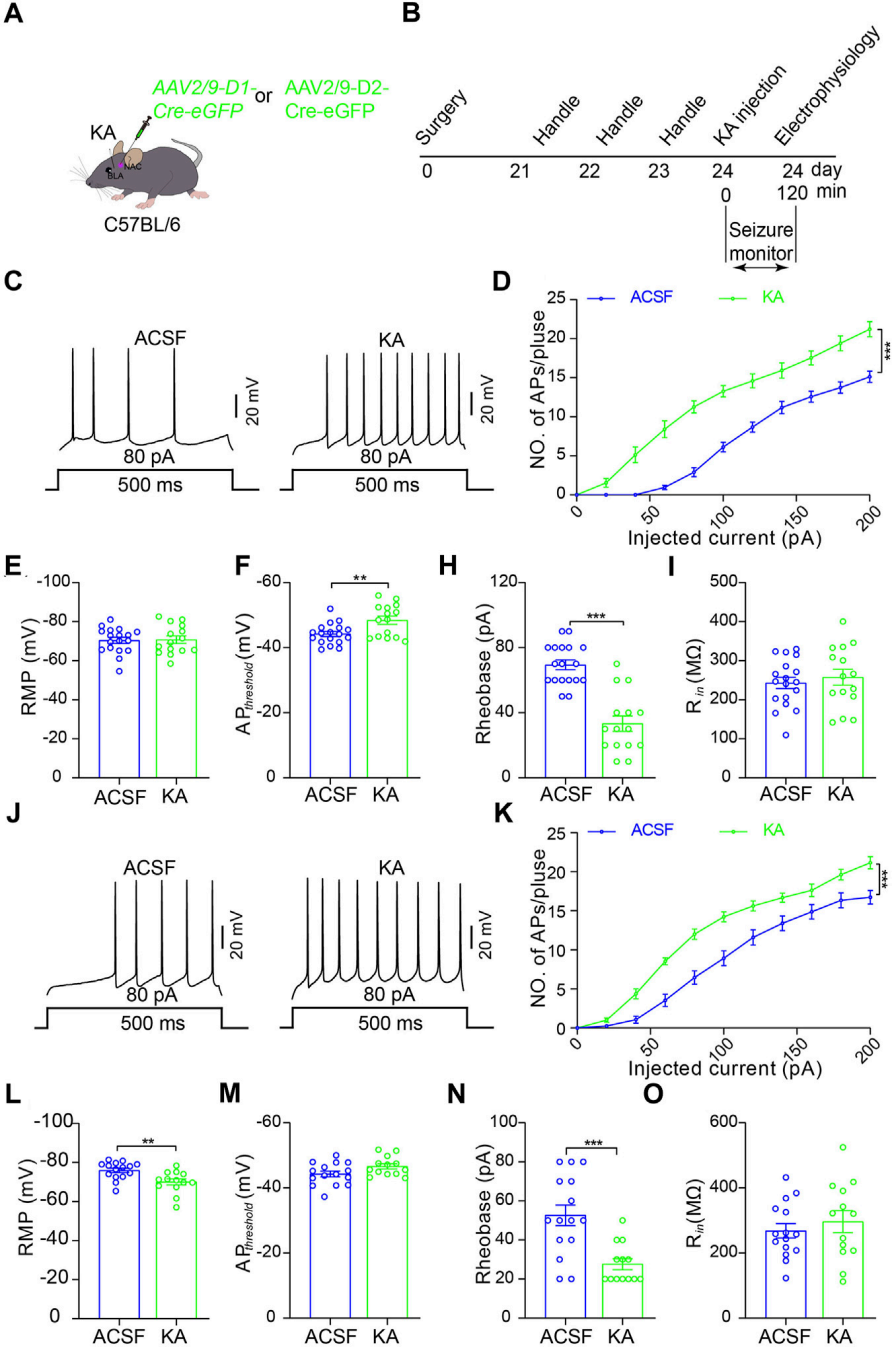
Increased excitability of D1R- and D2R-MSNs in the NAc shell after TLE seizures. **(A)** Schematic diagram of virus microinjection to enable expression of eGFP reporter on D1R-MSNs or D2R-MSNs in the NAc shell and KA microinjection into BLA to induce TLE seizures of C57BL/6 mouse. **(B)** Schematic diagram of TLE seizure induction followed by in intro electrophysiological recording in the NAc shell. **(C)** Representative current-clamp recordings. **(D)** Summary input–output curves of D1R-MSN of the mice treated KA (*n* = 15 cells) show increased AP generations in response to current injections, comparing with the control mice treated with ACSF (*n* = 18 cells). ***p < 0.001, F = 70.049, df = 1, two-way ANOVA with Tukey post-hoc test. **(E–I)** Summary data of resting membrane potential (RMP), AP threshold, rheobase current, and membrane input resistance (R_in_) of D1R-MSN of the mice treated with ACSF (*n* = 18 cells) or KA (*n* = 15 cells). **p < 0.01, ***p < 0.001, Student’s unpaired t-test. **(J)** Representative current-clamp recordings and **(K)** summary input–output curves of D2R-MSN of the mice treated KA (*n* = 13 cells) show increased AP generations in response to current injections, compared with the control mice treated with ACSF (*n* = 15 cells). ***p < 0.001, F = 20.136, df = 1, two-way ANOVA with Tukey post-hoc test. **(L–O)** Summary data of resting membrane potential (RMP), AP threshold, rheobase current, and membrane input resistance (R_in_) of D2R-MSN of the mice treated with ACSF (*n* = 15 cells) or KA (*n* = 13 cells). **p < 0.01, ***p < 0.001, student’s unpaired t-test.

**FIGURE 4:**
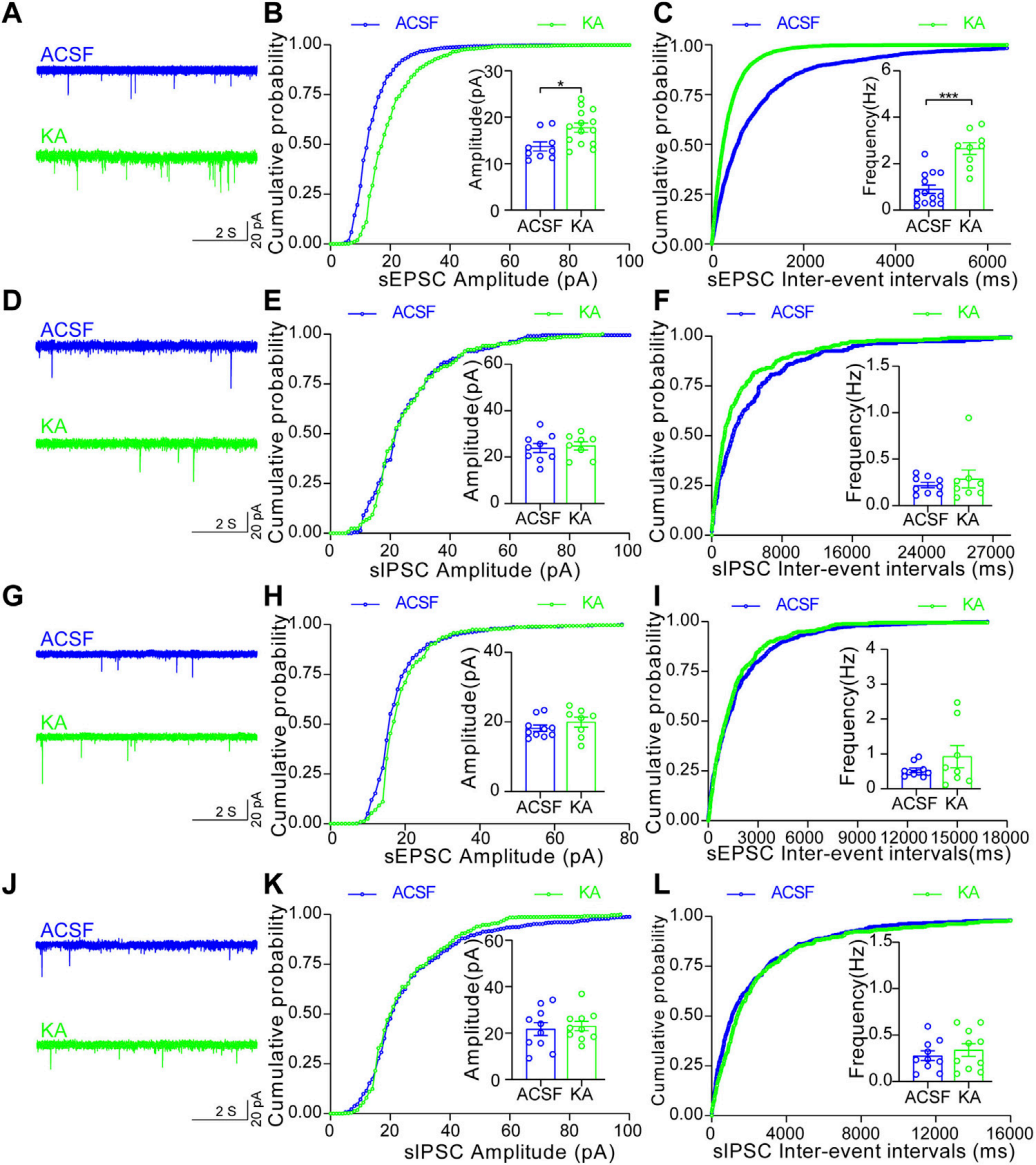
Increased synaptic transmission of D1R-MSN, but not D2R-MSN, in the NAc shell after TLE seizures. **(A)** Representative sEPSC of D1R-MSN of the mice treated with ACSF or KA. **(B)** Cumulative probability of the amplitudes and the averaged amplitude of sEPSCs of D1R-MSN of the mice treated with ACSF (*n* = 9 cells) or KA (*n* = 14 cells). *p < 0.05, Student’s unpaired t-test (the same test was used below in this figure). **(C)** Cumulative probability of the inter-event intervals of sEPSCs and the averaged frequency of sEPSCs of D1R-MSN of the mice treated with ACSF (*n* = 14 cells) or KA (*n* = 9 cells). ***p < 0.001. **(D)** Representative recordings of sIPSC of D1R-MSN of the mice treated with ACSF or KA. **(E)** Cumulative probability of the amplitudes and the averaged amplitude of sIPSCs of D1R-MSN of the mice treated with ACSF (*n* = 9 cells) or KA (*n* = 8 cells). **(F)** Cumulative probability of the inter-event intervals of sIPSCs and the averaged frequency of sIPSCs of D1R-MSN of the mice treated with ACSF (*n* = 9 cells) or KA (*n* = 8 cells). **(G)** Representative recordings of sEPSC of D2R-MSN of the mice treated with ACSF or KA. **(H)** Cumulative probability of the amplitudes and the averaged amplitude of sEPSCs of D2R-MSN of the mice treated with ACSF (*n* = 10 cells) or KA (*n* = 8 cells). **(I)** Cumulative probability of the inter-event intervals of sEPSCs and the averaged frequency of sEPSCs of D2R-MSN of the mice treated with ACSF (*n* = 10 cells) or KA (*n* = 8 cells). **(J)** Representative recordings of sIPSC of D2R-MSN of the mice treated with ACSF or KA. **(K)** Cumulative probability of the amplitudes and the averaged amplitude of sIPSCs of D2R-MSN of the mice treated with ACSF (*n* = 10 cells) or KA (*n* = 10 cells). **(L)** Cumulative probability of the inter-event intervals of sIPSCs and the averaged frequency of sIPSCs of D2R-MSN of the mice treated with ACSF (*n* = 10 cells) or KA (*n* = 10 cells).

To further examine which type of MSNs in the NAc shell is activated by TLE seizure activity, we performed electrophysiological recordings on D1R-MSN and D2R-MSN labeled by enhanced green fluorescence protein (eGFP) through the transfection of AAV2/9-D1-Cre-eGFP and AAV2/9-D2-Cre-eGFP, respectively, after seizure induction with intra-amygdala KA (Figures 3A,B). Compared with the ACSF-injected control mice, D1R-MSN of the KA-injected mice showed increased action potential (AP) firing and decreased AP threshold and AP induction rheobase (Figures 3C–I), while D2R-MSN of the KA-injected mice also showed increased AP firing and decreased AP induction rheobase (Figures 3J–O). Meanwhile, TLE seizures enhanced the amplitude and frequency of spontaneous excitatory postsynaptic currents (sEPSCs) of D1R-MSN (Figures 4A–C), but they neither changed the amplitude and frequency of spontaneous inhibitory postsynaptic currents (sIPSCs) of D1R-MSN (Figures 4D–F) nor changed the sEPSC or sIPSC of D2R-MSN (Figures 4G–L). These electrophysiological experiments in combination with the aforementioned experiments detecting cell-type-specific c-fos expression illustrate that both D1R-MSN and D2R-MSN in the NAc shell are activated in the process of TLE seizures, and specifically, excitatory synaptic transmission of D1R-MSN is enhanced by seizures.

### Pharmacological inhibition of the NAc shell reduces the propagation of TLE seizures

**FIGURE 5:**
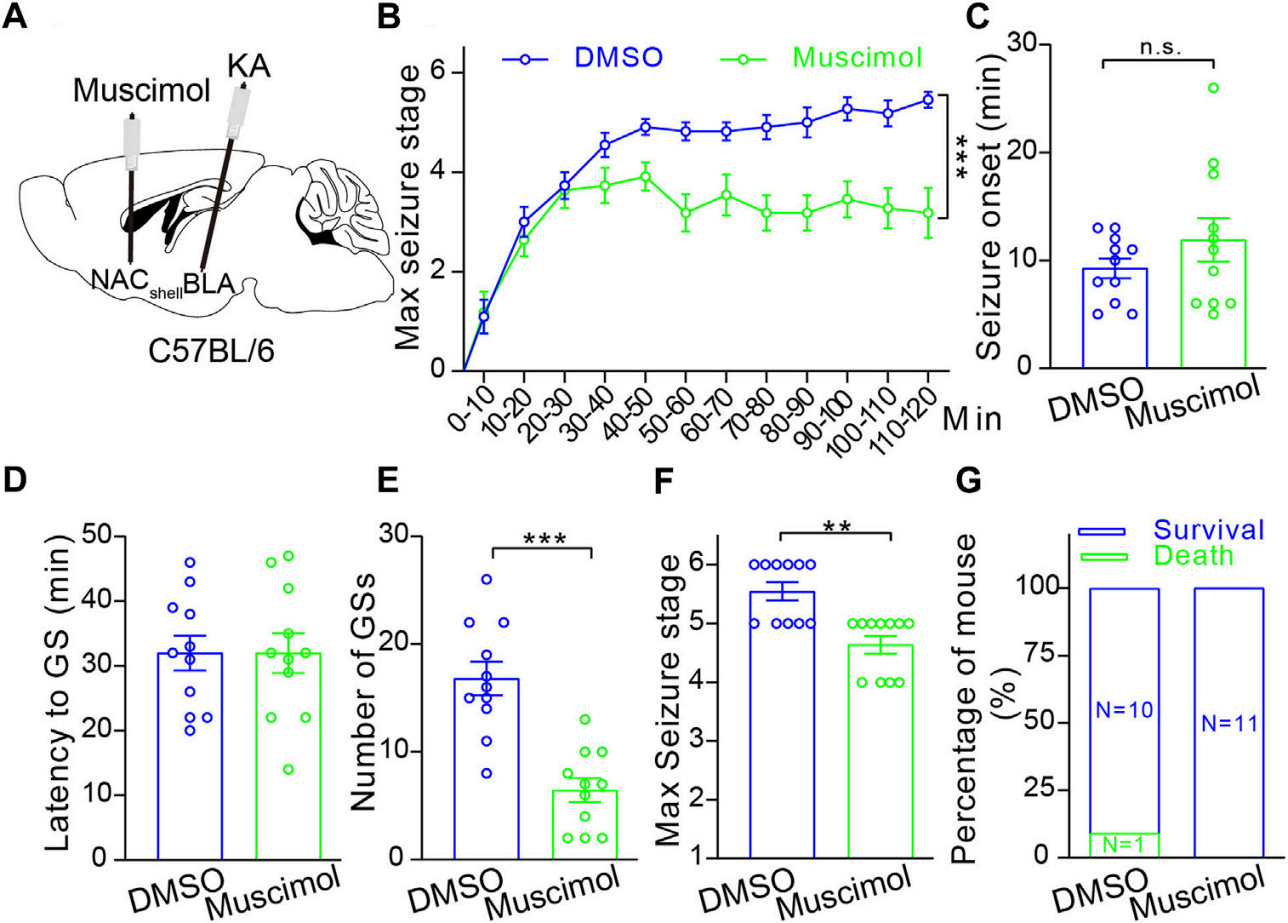
Pharmacological inhibition of the NAc shell alleviates TLE seizures. **(A)** Schematic diagram of muscimol microinjection to inhibit the NAc shell and 10 min later KA microinjection into BLA to induce TLE seizures of the C57BL/6 mouse. **(B)** Racine’s scaling of the mice treated with DMSO control (*n* = 11 mice) or muscimol (*n* = 11 mice), showing the maximal seizure stage every 10 min during the whole course of 120 min after KA microinjection. ***p < 0.001, two-way ANOVA with repeated-measures. **(C)** The onset time of the first seizure after KA microinjection. DMSO control: *n* = 11 mice; muscimol *n* = 11 mice; Student’s unpaired t-test. **(D)** Latency to secondarily general seizures (GSs) after KA microinjection. DMSO control: *n* = 11 mice; muscimol *n* = 11 mice; Student’s unpaired t-test. **(E)** Total number of secondarily GSs after KA microinjection. DMSO control: *n* = 11 mice; muscimol *n* = 11 mice; ***p < 0.001, Student’s unpaired t-test. **(F)** Maximal seizure stage in the whole course of 120 min after KA microinjection. DMSO control: *n* = 11 mice; muscimol *n* = 11 mice; **p < 0.01, Mann–Whitney rank sum Test. **(G)** Mortality rate after KA microinjection. DMSO control: *n* = 11 mice; muscimol *n* = 11 mice; chi-square test.

Then, to examine whether the NAc shell can modulate epileptic activity, we focally inhibited the NAc shell by microinjection of GABA_A_ receptor agonist muscimol (0.5 μM) 10 min before intra-amygdala induction of TLE seizures (Figure 5A). Under this condition, we found that inactivation of the NAc shell reduced the severity of seizures by lowering seizure stages (Figure 5B), reducing the occurrence of SGSs (Figure 5E) and the maximal seizure stage (Figure 5F) within 2 h following induction, but it did not alter the seizure onset which reflects the onset time of focal seizures (Figure 5C), the latency to SGSs (Figure 5D) and lethality (Figure 5G). Considering that the NAc shell is located outside of the temporal lobe and downstream to the BLA, these data indicate that the NAc shell participates in the propagation of TLE seizures induced by intra-amygdala KA.

### Chemogenetic inhibition of D1R-MSN or D2R-MSN in the NAc shell reduces propagation of TLE seizures

**FIGURE 6:**
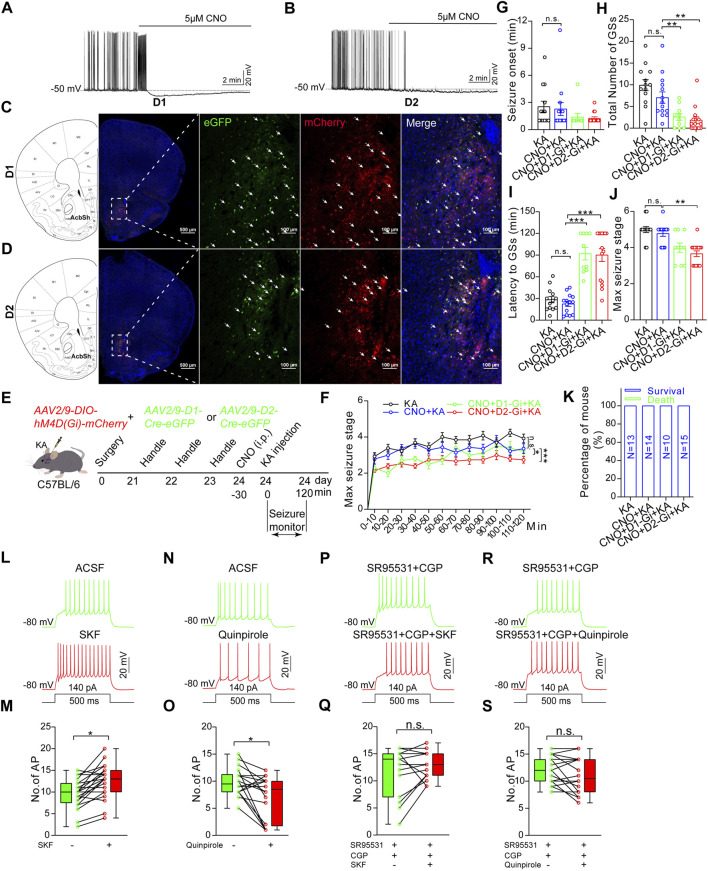
Chemogenetic inhibition of D1R-MSN or D2R-MSN in the NAc shell alleviates TLE seizures. **(A)** Representative recording shows spontaneous AP firings of D1R-MSN and **(B)** D2R-MSN transfected with DREADD receptor hM4D (Gi) are suppressed by CNO (5 μM) perfusion. Membrane potential was held under current-clamp at around −50 mV. **(C)** Representative confocal images show the expression of mCherry and eGFP on D1R-MSN transfected with AAV2/9-DIO-hM4D(Gi)-mCherry and AAV2/9-D1-Cre-eGFP viruses. **(D)** Representative confocal images show the expression of mCherry and eGFP on D2R-MSN transfected with AAV2/9-DIO-hM4D(Gi)-mCherry and AAV2/9-D2-Cre-eGFP viruses. **(E)** Schematic diagram of experimental design to examine the effects of chemogenetic inhibition of D1R-MSN or D2R-MSN in the NAc shell on TLE seizures induced by intra-basolateral amygdala (intra-amygdala) microinjection of KA. The mice are allocated into four groups: KA group *n* = 13 mice; CNO + KA group *n* = 14 mice; CNO + D1-Gi + KA group *n* = 10 mice; CNO + D2-Gi + KA group *n* = 15 mice. **(F)** Racine’s scaling for maximal seizure stage every 10 min during the whole course of 120 min after KA microinjection. *p < 0.05, ***p < 0.001, F = 18.46, df = 3, two-way ANOVA with repeated-measures. **(G)** Onset time of the first seizure after KA microinjection. One-way ANOVA. **(H)** Latency to secondary GSs after KA microinjection. **p < 0.01, one-way ANOVA. **(I)** Total number of secondary GSs after KA microinjection. ***p < 0.001, one-way ANOVA. **(J)** Maximal seizure stage in the whole course of 120 min after KA microinjection. **p < 0.01, Kruskal–Wallis H test with Bonferroni correction **(K)** Mortality rate after KA microinjection. *p* > 0.05, Kruskal–Wallis H test. **(L)** Representative recording and statistical data **(M)** show AP firing in response to a step current before or after perfusion of 15 μM D1R agonist SKF 38393. *n* = 21 cells, *p < 0.05, Student’s unpaired t-test. **(N)** Representative recording and statistical data **(O)** show AP firing in response to a step current before or after perfusion of 15 μM D2R agonist quinpirole. *n* = 18 cells, *p < 0.05, Student’s unpaired t-test. **(P)** Representative recording and statistical data **(Q)** show AP firing in response to a step current before or after perfusion of 15 μM D1R agonist SKF 38393 in the presence of GABA_A/B_ blockers (10 μM SR95531/100 μM CGP35348). *n* = 15 cells, *p* > 0.05, Student’s unpaired t-test. **(R)** Representative recording and statistical data **(S)** show AP firing in response to a step current before or after perfusion of 15 μM D2R agonist quinpirole in the presence of GABA_A/B_ blockers. *n* = 20 cells, *p* > 0.05, Student’s unpaired t-test. In the figures G–K, the statistical differences are compared between the KA group and the CNO + KA group, and between the CNO + KA group and the CNO + D1R-Gi + KA group or the CNO + D2R-Gi + KA group. n.s means not statistically significant.

Finally, we wanted to identify the roles of D1R-MSN and D2R-MSN in the modulation of TLE seizures, since D1R-MSN and D2R-MSN in the NAc shell are recruited by the seizures induced by intra-amygdala KA and pharmacological inhibition of the NAc shell suppresses propagation of the seizures. For this purpose, we delivered AAV vectors into the NAc shell to express the Gi-coupled chemogenetic DREADD receptor (designer receptor exclusively activated by designer drugs) hM4D (Gi) (AAV2/9-hysn-DIO-hM4D (Gi)-mcherry-WPRE-PA) in a Cre-dependent manner (AAV2/9-D1-Cre-eGFP or AAV2/9-D2-Cre-eGFP) on MSN. AP firing of both D1R-MSN and D2R-MSN that expressed Cre-dependent hM4D (Gi) was suppressed upon the perfusion of designer drug CNO (clozapine N-oxide, 5 μM) to the brain slice acquired 3 weeks after virus injection (Figures 6A,B) when the DREADD receptor was expressed with reporter fluorescent protein eGFP (Figures 6C,D), verifying the effective and functional expression of DREADD receptors. Evaluation of TLE seizures, which were induced by intra-amygdala KA primed with i.p. injection of CNO (4 mg/kg, Figure 6E), showed that the mice carrying hM4D (Gi), compared with the mice without carrying hM4D (Gi) on either D1R-MSN or D2R-MSN in the NAc shell, exhibited decreased seizure stages (Figures 6F,J) and number of the SGSs (Figure 5I), increased latency of the SGSs (Figure 6H), but similar seizure onset which reflects the onset time of focal seizures (Figure 6G) and the lethality (Figure 6K). Therefore, chemogenetic inhibition of either of the MSNs in the NAc shell reduces propagation of TLE seizures induced by intra-amygdala KA.

It is worth noting that no significance of any of those seizure-related parameters was observed when comparing the CNO-treated mice carrying hM4D (Gi) on D1R-MSN and D2R-MSN. This unexpected similar action between D1R-MSN and D2R-MSN could be due to consequential interaction directly or indirectly coupling the two MSN populations. We hypothesized that if interaction exists between the two populations of MSNs, preventing their electrophysiological output by agents blocking GABAergic synaptic transmission should alter the effects of selective activation of D1R or D2R on overall neuronal excitability of the NAc shell. To test this hypothesis, we recorded AP firings of MSNs in the NAc shell without knowing MSN subtypes and evaluated the average AP firings evoked by depolarizing step currents in the presence of specific D1R or D2R agonist (SKF 38393 and quinpirole), in comparison with baseline AP firings in the absence of the agonist. We found that D1R agonist significantly increased (AP number/0.5s: baseline 9.57 ± 0.74, SKF 12.52 ± 0.86, *n* = 21, *p* < 0.05. Figures 6L,M), while D2R agonist significantly decreased (AP number/0.5s: baseline 9.83 ± 0.61, quinpirole 7.11 ± 0.95, *n* = 18, *p* < 0.05. Figures 6N,O) the average AP firings of MSNs of unknown subtypes. When the slices were perfused with GABA_A/B_ receptor blockers (SR95531/CGP35348) to prevent the GABAergic output of MSNs, both D1R and D2R agonists failed to exert net effects on MSN AP firings (Figures 6P–S). These verify that under intact local circuits, D1R-MSN and D2R-MSN interplay directly or indirectly. Activation of D1R-MSNs can gain an overall enhanced neuronal activity, while that of D1R-MSNs may produce an overall suppressed neuronal activity in the NAc.

## Discussion

DBS of the NAc is considered as an alternative treatment for the refractory drug addiction. Interestingly, some clinical data provide preliminary indication that DBS of the NAc may have an anti-epileptic effect (Schmitt et al., 2014; Kowski et al., 2015). In TLE patients, the structural and connection abnormalities of NAc are mainly found in its shell proportion (Zhao et al., 2018; Yang et al., 2020; Zhu et al., 2021). In the Noda epileptic rats that exhibit TLE-like spontaneous generalized seizures, seizures evoke c-fos expression in the NAc shell and core (Ohno et al., 2009). In the rat model of TLE that is induced by i.p. injection of pentylenetetrazol or KA, the expression of c-fos and cyclooxygenase-2 increase in the NAc shell (Joseph et al., 2006; Szyndler et al., 2009). In the murine models of TLE, neuronal injury (Torolira et al., 2016) was observed and increased concentrations of amino acids including kynurenic acid (Baran et al., 1995; Loscher et al., 1996) and glutamate (Maciejak et al., 2010) were detected in the NAc. In the rhesus macaques with TLE, DBS to anterior nuclei of the thalamus decreased the amount of glutamate receptor 1 in the NAc shell (Du et al., 2021). These, together with some other studies (Richman and Heinrichs, 2007; Wolf et al., 2016), indicate that the NAc shell is strongly involved in the TLE, consistent with our data showing higher c-fos expression in the NAc shell of the epileptic animals compared with the vehicle control animals. Although we cannot exclude the involvement of the NAc core, we thereafter focused on the roles of the NAc shell in the modulation of TLE seizures.

In the NAc, 95% of neurons are D1R-MSN or D2R-MSN that receive dopaminergic inputs from the VTA and excitatory inputs from the hippocampus, amygdala, prefrontal cortex, and thalamus (Sundar et al., 2021), and they send the GABAergic outputs to the VTA, basal ganglia, thalamus, hypothalamus, etc. (Xu et al., 2020). Such circuit connections of the NAc enable it to play important roles in the process of TLE seizures, especially for the NAc shell as the shell receives denser innervations from hippocampus and amygdala compared with the core (Scofield et al., 2016). In line with this idea, researchers already reported that the NAc exhibits pathological electric activities associated with behavioral seizures kindled in the basal lateral amygdala of rats (Wlaz et al., 1994; Ebert et al., 1995) and that the NAc shell exhibits increased gamma oscillations in the hippocampal-kindled rats (Ma and Leung, 2010), indicating that NAc shell participates in the propagation of TLE seizures. In this research, we further provide direct evidence of NAc shell participating in the propagation of TLE seizures by our pharmacological experiment which shows inhibiting NAc shell with local microinjection of muscimol does not affect the onset of focal behavioral seizures, but diminishes the number of behavioral SGSs and relieves seizure stages of the intra-amygdala model of TLE.

Both D1R-MSN and D2R-MSN are GABAergic neurons, but they function differentially *via* distinct cellular signaling and circuit networks. At the cellular level, activations of D1R and D2R cause excitatory action on D1R-MSN through stimulatory G-protein (Gs) signaling and inhibitory action on D2R-MSN through inhibitory G-proteins (Gi), respectively. At the circuit level, D1R-MSN and D2R-MSN synapse each other to form local circuits. Thus, D1R-MSN and D2R-MSN can cause direct mutual suppressing effects on adjacent MSN, but D1R-MSN is able to drive excitatory action on D2R-MSN *via* indirect circuits in the NAc (Francis et al., 2019). As projection neurons, NAc D1R-MSN and D2R-MSN send bias innervation to other regions, for example, both D1R-MSN and D2-MSN give afferents to the ventral pallidum, but D1R-MSN selectively innervates the VTA (Pardo-Garcia et al., 2019). Those discrepancies endow D1R-MSN and D2R-MSN of the NAc distinct functions in modulating reward and aversion (Ikemoto et al., 1997; Danjo et al., 2014; Soares-Cunha et al., 2020), motivation (Ikemoto et al., 1997), drug seeking and goal-directed behaviors (Pezze et al., 2007; Lex and Hauber, 2008), mood (Francis and Lobo, 2017; Pena, 2017), wakefulness (Luo et al., 2018), etc. (Sato et al., 2022). However, cell-type-specific roles of NAc MSNs in the modulation of TLE seizures have not been investigated yet. In this research, we find that both types of MSNs in the NAc shell exert increased neuronal excitability and D1R-MSN exerts increased excitatory synaptic transmission after TLE seizure induced by intra-amygdala KA. In this TLE model, chemogenetic inhibition of either type of MSNs in the NAc shell or inhibiting the entire NAc shell pharmacologically with muscimol suppresses the propagation, but not initiation of TLE seizures, as both chemogenetic and pharmacological inhibition delays the development of generalized seizures and reduces SGSs, while both inhibitory methods showed no notable effect on the onset of focal seizures However, the NAc shell and its MSNs may play different roles in the seizure initiation, propagation, and termination in other epileptic models other than TLE.

D1R is coupled with Gs protein so that inactivation of D1R-MSN can be fulfilled through antagonizing D1R. On the contrary, D2R is coupled with Gi protein so that inactivation of D2R-MSN can be fulfilled through agonizing D2R. Controversial effects of dopaminergic agonists and antagonists applied in *in vitro* or systematic ways on seizures have been reported, but in most cases, it was reported that dopamine D1R agonists are proconvulsant and that D2R agonists are anticonvulsant (Wahnschaffe and Loscher, 1991; Starr, 1996; Sharopov et al., 2012; Brodovskaya and Kapur, 2021). In line with these previous studies showing D1R antagonists and D2R agonists are antiepileptic, our results of chemogenetic inhibition of either D1R-MSN or D2R-MSN in the NAc shell proves to be protective for the brain from more severe seizure insult. Interestingly, one research in the early 1990s revealed that focal administration of D2R agonist LY 171555 into the NAc is enough to provide antiepileptic effects in the amygdala-kindled epilepsy model (Wahnschaffe and Loscher, 1991). In this study, chemogenetic inhibition of D2R-MSN in the NAc shell also produced antiepileptic effects. The consistency between this research and the previous one (Wahnschaffe and Loscher, 1991) could be in that the agonist-induced activation of D2R also causes inactivation of D2R-MSN as D2R is coupled with inhibitory G protein and in that the modulatory effect of NAc on TLE seizures mainly depends on the shell portion.

Even more, our data investigating functional interaction between D1R-MSN and D2R-MSN revealed that activation of the two populations of MSNs influences each other. Agonist-induced activation of D1R-MSNs gains an overall enhanced neuronal activity, while that of D2R-MSNs produces an overall suppressed neuronal activity in the NAc, supporting the notion that lowering the overall NAc shell excitability by antagonizing D1R-MSNs or agonizing D2R-MSNs are antiepileptic in view of TLE seizures propagating through the NAc shell. Besides, poorly controlled SE is one of the highest risk factors for chronic epileptogenesis (Tong et al., 2022). Repetitive propagation of TLE seizures leads to additional brain damages in brain regions outside of initial focus which give rise to secondary epileptogenesis and comorbidities (Dhaher et al., 2021). Thus, interfering with the NAc shell neurons of the TLE brain may not only confine acute seizure severity, but also may limit the development of drug-resistant epileptogenesis and related neurological comorbidities at the post-SE stages.

## Conclusion

Our data revealed that the NAc Shell and its MSNs are activated by TLE seizures. Both D1R-MSN and D2R-MSN in the NAc shell participate in the propagation of TLE seizures. Although the antiseizure effects of chemogenetic inhibition of D1R-MSN or D2R-MSN are modest and do not warrant their use as a potential antiepileptic strategy, enhancing inhibitory tone by DBS, dopaminergic ligands, and antiepileptic medications precisely on D1R-MSN or D2R-MSN could be the future antiepileptic strategy.

## Methods

### Animals

The animals were housed in a plastic cage (300 × 170 × 120 mm) at a standard laboratory animal environment (12 h light-dark cycle, temperature at 23 ± 1°C, and humidity at 40%) with free access to food and water. The C57BL/6J mice were purchased from the Guangdong Medical Laboratory Animal Center. The Drd1-iCre mice were obtained from Gempharmatech Co., Ltd.; the Drd2-Cre mice were obtained from the Mutant Mouse Resource and Research Center (MMRRC); and the Ai14 mice were obtained from the Jackson Laboratory. The mice used in this study were at the age of 8–12 weeks and to avoid effects of female hormones and circle on behaviors only male mice were used. All behavioral tests were conducted during the period of light.

### Epileptic models and evaluation of seizures

In the intra-amygdala KA (kainic acid) model, the mice were stereotactically implanted with a guide cannula into the right basolateral amygdala (BLA) nucleus (coordinates from bregma: AP = −0.9 mm, ML = −3.12 mm, DV = −5.0 mm with respect to skull surface) and fixed with dental cement under anesthesia with pentobarbital sodium (50 mg/kg). After recovery for 7–10 days, KA (0.2 µg in 0.2 µl ACSF, artificial cerebrospinal fluid) was micro-injected into amygdala through an injection cannula connected to a 5-µl Hamilton syringe (# 65460-02, 5 µl). In the lithium chloride (LiCl)–pilocarpine model of epilepsy, the mice were intraperitoneally (i.p.) injected with LiCl (10 mEq/kg, Sigma #213233) 20 h prior to i.p. injection of pilocarpine (60 mg/kg, MedChemExpress #HY-B0726). To reduce the peripheral effects of pilocarpine, the mice were given an i.p. injection of scopolamine methyl nitrate (1 mg/kg, Sigma #S2250) 30 min before pilocarpine injection. The control mice received vehicle injection.

Behavioral seizure severity in response to intra-amygdala KA injection was assessed by Racine’s standard clssification into 0–6 stages (Racine, 1972a;b): 0) no abnormality; 1) eye blinking, mouth, and/or facial movements; 2) head nodding; 3) unilateral forelimb clonus; 4) rearing with clonus; 5) loss of posture; and 6) status epilepticus (SE, continuous seizure activity lasting at least 30 min) and death. Epileptic seizures were scored for the maximal stage every 10 min for 120 min following KA injection. Seizure latency was the time from KA injection to the first focal or generalized seizures. Focal and secondarily generalized seizures are defined as stages 1–3 (Racine, 1972a) and stages 4–6 seizures (Racine, 1972b), respectively. At the end of seizure monitoring, seizures were terminated by pentobarbital sodium (25 mg/kg, i.p.). Some mice were killed under anesthesia and subjected to immunofluorescence staining after behavioral evaluation.

### Electrophysiology

Mice were anesthetized with pentobarbital sodium (50 mg/kg) and then quickly decapitated to remove the brain into the ice-cold cutting solution which contains (in mM) 2 KCl, 1.25 NaHPO_4_, 0.2 CaCl_2_, 12 MgSO_4_, 10 D-Glucose, 220 Sucrose, and 26 NaHCO_3_ and is oxygenated with 95% O_2_ + 5% CO_2_. Coronal brain slices including the nucleus accumbens (300 μm) were prepared with vibratome (VT1200S, Leica). After sectioning, the slices were incubated in standard ACSF which contains (in mM) 126 NaCl, 2.5 KCl, 1.25 NaHPO_4_, 2 CaCl_2_, 10 D-Glucose, and 26 NaHCO_3_ and is oxygenated with 95% O_2_ + 5% CO_2_ for 20–30 min at 34°C. Then, the slices were allowed to recover for at least 1 h at room temperature before recording. Whole-cell path-clamp recordings were performed using a Multi-Clamp 700 B amplifier, with signals being recorded/analyzed using the Digidata 1440 A data acquisition system and pClamp 10.7 software package (Molecular Devices). Recordings were conducted on medium spiny neurons (MSN) visually identified by fluorescence of reporter proteins in the nucleus accumbens shell (NAc shell) region using patch pipettes, which had a resistance of 4–6 MΩ and were filled with intracellular solutions. Spontaneous excitatory postsynaptic currents (sEPSCs) were recorded under voltage clamp conditions with pipette solution which contains (in mM) 135 K-Gluconate, 5 KCl, 10 HEPES, 2 MgCl_2_, 0.2 Na_2_-ATP, and 2 Mg-ATP and holding potential at −70 mV in ACSF containing 10 μM SR95531 (sigma #S106) to block GABA_A_ receptors. Spontaneous inhibitory postsynaptic currents (sIPSCs) were recorded under voltage clamp conditions with pipette solution which contains (in mM) 110 Cs_2_SO_4_, 0.5 CaCl_2_, 2 MgCl_2_, 5 EGTA, 5 HEPES, 5 TEA, and 5 Mg-ATP and holding potential at −70 mV in ACSF containing 50 μM AP-5 (Sigma #A5282) and 20 μM CNQX (sigma #C127) to block glutamate receptors. To characterize neuronal intrinsic properties, the cells were current-clamped with the same pipette solution used in recording the sEPSCs. In the experiments investigating functional interaction between D1R-MSN and D2R-MSN, 15 μM SKF 38393 hydrochloride (MedChemExpress #HY-12520 A) was used to activate D1R, 15 μM quinpirole hydrochloride (sigma # Q102) was used to activate D2R, and 100 μM CGP35348 (MedChemExpress #HY-103530) was used to block GABA_B_ receptors.

### Microinjection of viruses and muscimol

To selectively identify medium spiny neurons (MSNs) expressing dopamine D1 receptor (D1R) or D2 receptor (D2R) for the electrophysiological study, the C57 mice (at the age of 8–12 weeks) were stereotactically injected with AAV2/9-D1-Cre-eGFP or AAV2/9-D2-Cre-eGFP,. To specifically inhibit the activation of D1R-MSN or D2R-MSN with chemogenetic approach, the C57 mice (postnatal weeks 7–8) were stereotactically injected with a mixture of rAVV2/9-Ef1a-DIO-hM4Di-mCherry-WPRE-pA (viral titer: 2.01*10^12^ v.g./ml) and rAAV2/9-D1-Cre-eGFP-WPRE-pA or rAAV2/9-D2-Cre-eGFP-WPRE-pA (viral titer: 3.44*10^12^ v.g./ml) in 2:1. All the viruses were designed and packaged by BrainVTA (BrainVTA, Co., Ltd., Wuhan, China) and were preserved at −80°C before use. For virus injection, the mice (at the age of 8–12 weeks) were deeply anesthetized with pentobarbital sodium (50 mg/kg) and fixed on a stereotaxic frame (RWD Life Science Co., LTD.), the viruses were delivered into the NAc shell bilaterally (AP: +1.80 mm; ML: ±0.75 mm; DV: −4.2 mm, 0.15 µl per site) at a rate of 150 nl/min with syringe controlled by a mini-pump. The syringe was maintained at least 5 min before retraction. After retraction of the syringe, the mice were recovered under a heated blanket. The viruses were allowed to express for 3–4 weeks.

To inhibit the NAc shell with pharmacological approach, the mice (at the age of 8–12 weeks) were deeply anesthetized with pentobarbital sodium (50 mg/kg) and fixed on stereotaxic to implant with a bilateral steel guide cannula in the NAc shell. The mice’s scalp was cut, and the whole skull was exposed. The cannula was targeted to the NAc shell (AP: +1.80 mm; ML: ±0.75 mm; DV: 4.2 mm). After surgery, the mice were allowed to recover for at least 7 days and then subjected to microinfusion. Muscimol was infused into the NAc shell bilaterally (0.5 μM in 150 nl 1% DMSO each site). The microinjection lasted 2 min, and then the needle was kept in the cannula for 5 min to allow the solution to diffuse in to the injection site and prevent the liquid leakage. In the case of dual infusions of both muscimol and KA, muscimol was infused 10 min before KA. Cannula location and viral expression were histologically verified after the behavioral studies. Only the mice with correct locations of the cannula and viral expression were taken into analysis.

### Immunofluoresensence staining

Mice were killed under anesthesia with pentobarbital sodium (75 mg/kg, i.p.). The brain slices were sectioned at 40 μm and were rinsed in phosphate-buffered saline (PBS). After washing the residual embedding medium (OCT), the slices were blocked by 5% bovine serum albumin (BSA) with 1% Triton-X 100 for 1.5 h, and then incubated with anti-c-fos (1:1000, Millipore #ABE457) in 5% BSA overnight at 4°C. After washing, the slices were incubated with Alexa Fluor 488 (1:500, ZSGB-Bio #ZF-0511) or 594 (1:500, ZSGB-Bio #ZF-0513) goat anti-rabbit or goat anti-mouse secondary antibody for 1.5 h, and then incubated with DAPI (1 μg/ml, Sigma #D9542) for 15 min at room temperature, washed three times with PBS for 15 min, and mounted onto slides. The slices were dried and mounted by coverslips with mounting medium. The samples were imaged using a Nikon A1R confocal microscope. Cell counting and fluorescent area calculation were performed by Imaris (version 9.0.1).

### Statistical analysis

Statistical analysis was performed by IBM SPSS 22.0. The differences were analyzed by Student’s t-test, the Mann–Whitney rank sum test, the ANOVA with Tukey post-hoc test, the chi-square test, or the Kruskal–Wallis H test with Bonferroni correction. Data are presented as mean ± SEM. Statistical significance is defined as *p* < 0.05. Figures were plotted using Graphpad Prism 8.0.

## Data Availability

The original contributions presented in the study are included in the article/Supplementary Material; further inquiries can be directed to the corresponding authors.

## Fundings

This work was supported by the grant from the National Natural Science Foundation of China (NSFC) (31771127), and Guangdong Science and Technology Department (2018B030334001, 2018B030340001, and 2018B030335001). All sources of funding received for the research has been submitted.
